# Surrogate endpoints for overall survival in randomised controlled trials of localised osteosarcoma: A meta-analytic evaluation

**DOI:** 10.1038/s41598-020-65591-z

**Published:** 2020-05-22

**Authors:** Kazuhiro Tanaka, Masanori Kawano, Tatsuya Iwasaki, Shogo Matsuda, Ichiro Itonaga, Hiroshi Tsumura

**Affiliations:** Department of Orthopaedic Surgery, Faculty of Medicine, Oita University, Oita, Japan

**Keywords:** Sarcoma, Surgical oncology

## Abstract

Event-free survival (EFS) is considered the most reliable surrogate endpoint for overall survival (OS) in randomised controlled trials (RCTs) of adjuvant therapies for malignant tumours. However, the surrogacy of intermediate endpoints such as EFS for OS in trials of patients with osteosarcoma has not been investigated to date. In this study, we investigated the correlation between OS and intermediate endpoints in RCTs of localised osteosarcoma. A systematic search identified 20 relevant RCTs. The correlations between the surrogate endpoints and OS were evaluated using weighted linear regression analyses and by calculating the Spearman rank correlation coefficients (ρ). The strength of the correlation was determined by calculating the coefficient of determination (R^2^). A total of 5,620 patients were randomly assigned to 45 treatment arms in the eligible 20 RCTs. The correlation between the hazard ratios for EFS and OS was moderate (R^2^ = 0.456, ρ = 0.440); this correlation tended to be weaker for patients with localised osteosarcoma excluding the patients with metastases. Overall, the trial-level correlation between the surrogate endpoints and OS was not robust in RCTs of osteosarcoma published to date. Hence, the suitability of the intermediate endpoints as surrogates for OS could not be confirmed.

## Introduction

Osteosarcoma is the most frequently diagnosed primary malignant bone tumour, with an annual incidence of approximately 800 patients in the United States^1^, and 200 in Japan^2^. The prognosis of patients with osteosarcoma has dramatically improved following the introduction of multi-drug combination chemotherapy regimens. The current standard chemotherapeutic agents for osteosarcoma include methotrexate, doxorubicin (Adriamycin), and cisplatin (the MAP regimen) as well as ifosfamide (IFM)^3^.

Histological response to preoperative chemotherapy is a known prognostic factor for patients with osteosarcoma. Patients who are good responders (i.e., those who exhibit ≥90% necrosis in the resected tumour specimen) have been shown to have better prognoses than those who are poor responders (i.e., <90% necrosis)^4^, although trials aimed at determining the optimal combination chemotherapy regimen for the latter group have been ongoing. The EURAMOS1 trial, which is the largest randomised controlled trial (RCT) for osteosarcoma to date, found no significant difference in the outcomes of patients treated with MAP alone versus those treated with MAP plus IFM and etoposide. As such, the investigators recommended MAP alone as the standard regimen for osteosarcoma, including in the poor responders^5^. In Japan, however, IFM is combined with MAP as postoperative chemotherapy for patients who respond poorly to MAP preoperatively^6^; randomised phase 3 trials are currently underway to verify the effectiveness of this combination regimen^7^.

Improvement of overall survival (OS) is the most important goal when treating patients with malignant tumours. OS can be clearly defined, as it is the final, immutable endpoint, and is therefore preferred in clinical trials investigating most malignant tumours. However, there are limitations when using OS as a primary endpoint, as it requires more number of patients and additional follow-up involving more time and effort as well as higher costs. Moreover, the emergence of effective new drugs might prolong the post-progression survival and OS, and the effects of the protocol and post-protocol treatments may overlap, thereby influencing OS, making the interpretation of RCT results difficult. Hence, a number of RCTs of patients with localised osteosarcoma have used the intermediate measure of event-free survival (EFS) as their primary endpoint, as this avoids the abovementioned limitations. However, adopting EFS as the primary endpoint requires strong evidence of its correlation with OS; yet, the suitability of EFS as a surrogate endpoint for OS in RCTs of localised osteosarcoma has not been verified. To that end, we conducted a meta-analysis of all published RCTs of localised osteosarcoma to investigate the suitability of the intermediate endpoints including EFS and pathological response rate (RR) as surrogates for OS.

## Methods

### Study selection and data extraction

A systematic search of PubMed, Scopus, EBSCOhost MEDLINE, and the Cochrane Central Register of Controlled Trials was conducted according to the ‘Preferred Reporting Items for Systematic Reviews and Meta-Analyses’ guidelines^8^. The search was conducted for all English-language RCTs related to chemotherapy for localised osteosarcoma that were published between January 1974 and July 2019. Eligible studies were RCTs of chemotherapy for newly diagnosed localised osteosarcoma. Non-randomised clinical trials, reviews, and meta-analyses were excluded. The clinical trials extracted by the search were screened and cross-checked independently by two authors.

The extracted data included the publication date, trial name, study phase, patient enrolment period, number of patients, sex, age, number of metastatic diseases, neo-adjuvant or adjuvant regimens, drugs and doses in the standard and experimental arms, primary and secondary endpoints, intention-to-treat (ITT) analysis, post-protocol treatment, pathological response to chemotherapy, survival, and adverse events. A phase 2/3 study was considered as phase 3 for the purposes of this analysis. The medians, hazard ratios (HRs), 95% confidence intervals (CIs), and *P*-values for both EFS and OS were extracted; disease-free survival was considered as the same as EFS in this study. The pathological RR was defined as the proportion of assessed patients with a complete or partial response based on the criteria of each study. Data on 1-year, 3-year, and 5-year EFS and OS rates were extracted based on Kaplan-Meier estimates; when these data were not described, Kaplan-Meier plots for EFS or OS were constructed for their estimation as binary proportions. The variances were calculated according to the methods guided by the Cochrane^9,10^. Data were extracted and crosschecked by two authors. In the event of discrepancies, a third author arbitrated to reach a consensus.

### Statistical analysis

The EFS and OS values were obtained by meta-analyses using pooled HRs and their corresponding 95% CIs. The odds ratios (ORs) and corresponding 95% CIs for 1-, 3-, and 5-year EFS and OS were also calculated. The meta-analyses were performed using inverse variance and a Mantel-Haenszel random- or fixed-effects model^11^. If the *P*-value in the heterogeneity test was less than 0.1, the random-effects model was applied. Cochrane’s Q-test and I2 statistics were used to evaluate heterogeneity. Meta-analyses were completed using the Review Manager software (version 5.3; Nordic Cochrane Centre, Cochrane Collaboration, Copenhagen, Denmark).

The association of HR for OS and HRs or ORs for surrogate endpoints was assessed to evaluate the trial-level surrogacy. A weighted least-square regression for log (HR for OS) and log (HR for EFS) was used for the evaluation of the relationship between OS and EFS with weights equal to the sample size of the trial^12,13^. The coefficient of determination (R^2^) was used to evaluate the strength of the association. The bootstrapping was used to estimate the 95% CIs of the R^2^ surrogacy measures. The nonparametric Spearman’s rank correlation coefficients (ρ) were also used to evaluate the correlation between surrogate endpoints and OS.

Coefficient values were assessed as follows; >0.9, excellent; >0.75, very good; >0.5, good; >0.25, moderate; and ≤0.25, poor^14^. Sensitivity analyses were performed by excluding studies that comprised patients with metastatic disease or those whose treatment arms included radiation therapy and immuno-stimulants from the evaluation of surrogacy. The statistical analyses were completed using SAS (version 9.4; SAS institute, Cary, NC, USA). *P*-values reflected two-sided tests, with *P* < 0.05 indicating statistical significance.

## Results

### Characteristics of the eligible RCTs

**Figure 1:**
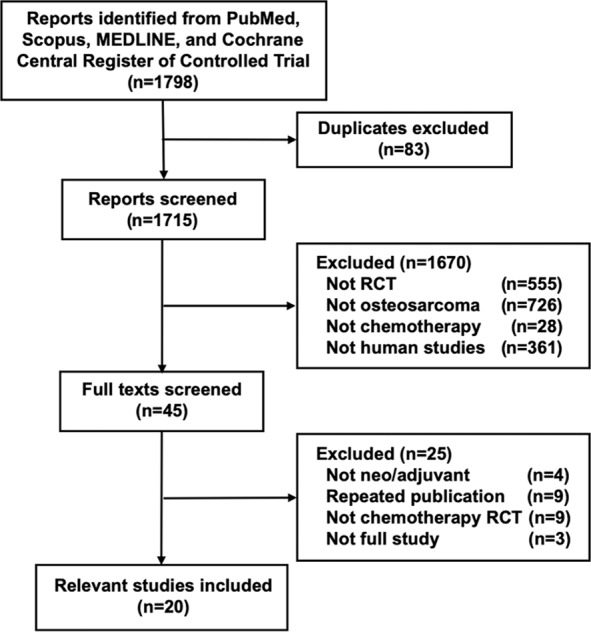
PRISMA flow diagram. PRISMA, Preferred Reporting Items for. Systematic Reviews and Meta-Analyses; RCT, randomised controlled trial.

Our systematic search of the literature identified 1,798 articles; 83 duplicate publications were excluded and remaining 1,715 studies were screened further. The full texts of 45 studies were finally evaluated after excluding 1,670 publications. Among these, nine repeat publications, nine non-chemotherapeutic studies, four non-neo-adjuvant or non-adjuvant setting studies, and three publications describing incomplete studies were further excluded. The remaining 20 RCTs were incorporated into the present analysis (Fig. 1, Table S1)^5,15–33^. The characteristics of the eligible RCTs are summarised in Table 1.

**Table 1:** Characteristics of the randomized controlled trials.

	No. of studies (%)	No. of patients (%)	Median no. of patients
	20 (100)	5620 (100)	198
**Trial phase**			
3	6 (30.0)	3028 (53.9)	561
Not specified	14 (70.0)	2592 (46.1)	142
**Primary endpoint**			
OS	2 (10.0)	709 (12.6)	354.5
EFS	9 (45.0)	3067 (54.6)	296
RR	3 (15.0)	701 (12.5)	188
Not specified	6 (30.0)	1143 (20.3)	160.5
**ITT analysis included**			
Yes	7 (35.0)	3074 (54.7)	391
No	13 (65.0)	2546 (45.3)	134.5

Abbreviations: EFS, event-free survival; ITT, intention-to-treat; OS, overall survival; RR, response rate.

The 5,620 patients whose data were extracted from the 20 eligible RCTs were randomly assigned to 45 treatment arms. All arms comprised combination chemotherapy regimens with two to eight cytotoxic drugs except one arm whose subjects underwent radiation alone and another whose patients underwent combination chemotherapy and bilateral lung irradiation^18^. No study involved administering a molecular targeted therapy, while two studies involved immuno-stimulants as protocol treatments^27,31^. Chemotherapy was administered in a neo-adjuvant setting in 37 treatment arms in 17 studies, and in an adjuvant setting in 7 treatment arms in 4 studies. Nine and three studies defined their primary endpoints as EFS and RR, respectively; only two RCTs used OS. Among the 20 RCTs, 5 involved patients with metastatic disease (n = 253 in total). The median EFS and OS were not reached in 14 (of 20) and 10 (of 13) RCTs, respectively; therefore, median survival analyses were not conducted in our study. Secondary malignant neoplasms were considered as events for EFS in 8 RCTs, and the total number of the patients that developed secondary malignancies was 36 (1.1%) out of 3,202.

**Figure 2:**
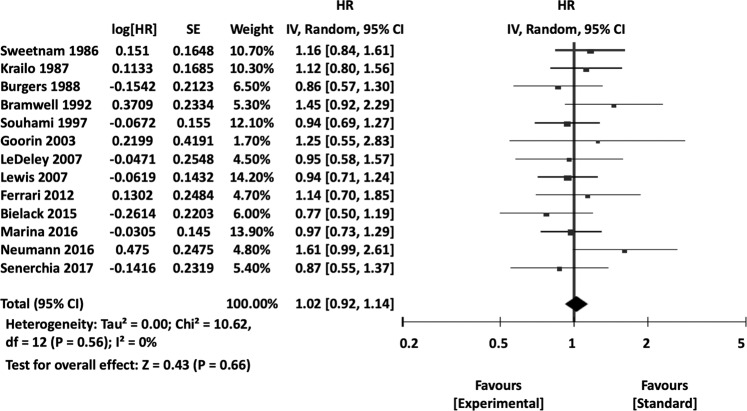
Forest plots of OS with comparisons of standard vs experimental chemotherapy. CI, confidence interval; HR, hazard ratio; IV, inverse variance; OS, overall survival; SE, standard error.

The HRs for EFS and OS were 1.01 (95% CI 0.92, 1.11, *P* = 0.78) and 1.02 (95% CI 0.92, 1.14, *P* = 0.66) (Fig. 2 and S1). There were no significant differences between the standard and experimental arms in terms of time-to-event endpoints, RRs, and severe adverse events (Table 2).

**Table 2:** Summary of meta-analyses.

Endpoint	HR/OR (95% CI)	*P*-value	No. of studies
EFS	1.01 (0.92, 1.11)	0.78	20
1-year EFS	0.83 (0.67, 1.02)	0.07	19
3-year EFS	1.05 (0.92, 1.20)	0.47	19
5-year EFS	1.06 (0.91, 1.23)	0.46	19
OS	1.02 (0.92, 1.14)	0.66	13
1-year OS	0.99 (0.75, 1.30)	0.92	13
3-year OS	1.00 (0.84, 1.19)	1.00	13
5-year OS	1.02 (0.89, 1.17)	0.75	13
RR	1.16 (0.81, 1.66)	0.40	11
AEs, overall	1.02 (0.59, 1.74)	0.95	10
Nausea/vomiting	0.56 (0.27, 1.16)	0.12	4
Leukopenia	0.82 (0.40, 1.68)	0.58	9

Abbreviations: AE, adverse event; CI, confidence interval; EFS, event-free survival; HR, hazard ratio; OR, odds ratio; OS, overall survival; RR, response rate.

### Association between intermediate endpoints and OS

Neither of the investigated intermediate endpoints showed excellent or very good association with the HR of OS. To assess the correlation between surrogate endpoints and OS, a weighted least-square regression model was fitted for the trial-level HRs, weighted with the sample size of the trial. For example, the linear regression equation of HR for EFS was as follows;

$$\log ({\rm{HR}}\,{\rm{for}}\,{\rm{OS}})=-\,0.024+0.750\,\times \,\log ({\rm{HR}}\,{\rm{for}}\,{\rm{EFS}}).$$

**Figure 3:**
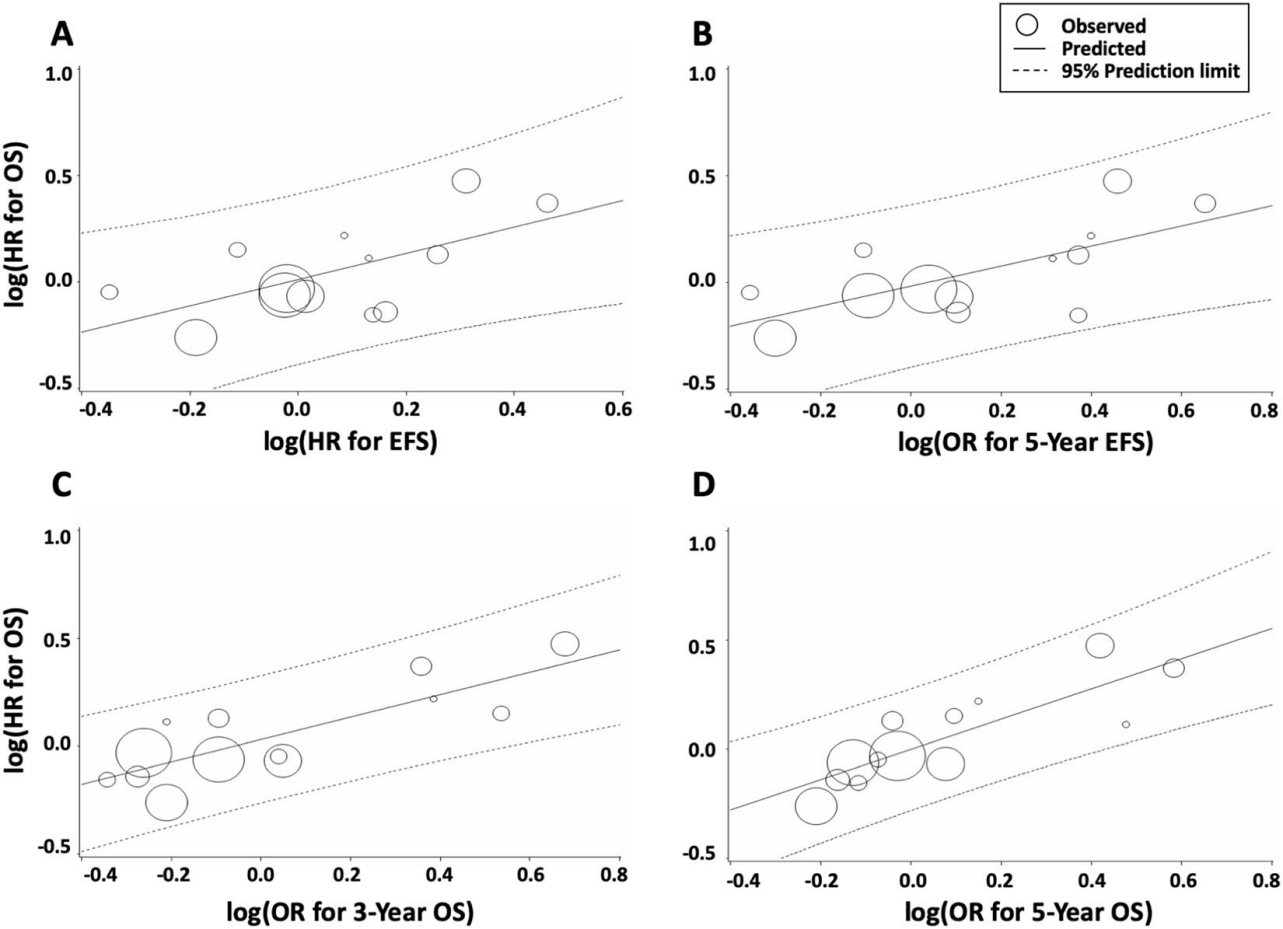
Correlation of intermediate endpoints with the HR for OS in patients with osteosarcoma. Correlation of (**A**) EFS HR, (**B**) 5-year EFS, (**C**) 3-year OS, and (**D**) 5-year OS. EFS, event-free survival; HR, hazard ratio; OR, odds ratio; OS, overall survival.

The trial-level correlation between the HRs of EFS and OS was moderate (R^2^ = 0.456, 95% CI 0.112, 0.799) (Fig. 3A and Table 3). The Spearman’s rank correlation coefficient (ρ) between the HRs of EFS and OS was 0.440 (95% CI −0.147, 0.797, *P* = 0.13). The association with the HR of OS was assessed as good (i.e., the coefficient of determination was >0.5) for the 5-year EFS (R^2^ = 0.530, 95% CI 0.210, 0.850; ρ = 0.564, 95% CI 0.019, 0.851, *P* = 0.0447), 3-year OS (R^2^ = 0.647, 95% CI 0.328, 0.912; ρ = 0.758, 95% CI 0.355, 0.923, *P* = 0.0027), and 5-year OS (R^2^ = 0.745, 95% CI 0.540, 0.951; ρ = 0.841, 95% CI 0.540, 0.951, *P* = 0.0003) (Fig. 3B–D and Table 3). On the other hand, the R^2^ for the association between the HR of OS and pathological RR was poor (R^2^ = 0.242, 95% CI 0.00, 0.709; ρ = −0.464, 95% CI −0.927, 0.558, *P* = 0.3542).

**Table 3:** Correlations between surrogate endpoints and OS in patients with osteosarcoma.

Surrogate endpoint	R^2^ (95% CI)	ρ (95% CI)	*P*-value for ρ	No. of studies
EFS	0.456 (0.112–0.799)	0.440 (−0.147, 0.797)	0.1329	13
1-year EFS	0.088 (0.00–0.341)	0.049 (−0.516, 0.585)	0.8725	13
3-year EFS	0.279 (0.00–0.634)	0.484 (−0.092, 0.817)	0.0941	13
5-year EFS	0.530 (0.210–0.850)	0.564 (0.019, 0.851)	0.0447	13
1-year OS	0.406 (0.052–0.759)	0.564 (0.019, 0.851)	0.0447	13
3-year OS	0.647 (0.382–0.912)	0.758 (0.355, 0.923)	0.0027	13
5-year OS	0.745 (0.540–0.951)	0.841 (0.540, 0.951)	0.0003	13
RR	0.242 (0.00–0.709)	−0.464 (−0.927, 0.558)	0.3542	6

Abbreviations: CI, confidence interval; EFS, event-free survival; OS, overall survival; RR, response rate.

Next, we conducted sensitivity analyses to evaluate the surrogacy after excluding the five studies that involved patients with metastatic disease. The coefficients of determination for EFS and OS in studies of localised osteosarcoma tended to be lower than those in all studies together. The correlation between the HRs of EFS and OS was poor (R^2^ = 0.156, 95% CI 0.00, 0.511; ρ = 0.083, 95% CI −0.615, 0.708, *P* = 0.8312) (Table 4). The association between the HR of OS and RR in studies of localised osteosarcoma alone was good (R^2^ = 0.598, 95% CI 0.075, 1.00); however, the correlation was inverse and not significant (ρ = −0.800, 95% CI −0.996, 0.697, *P* = 0.20). Further sensitivity analyses to evaluate the surrogacy after excluding the three studies that involved radiation therapy and immuno-stimulants in the treatment arms were performed. The coefficients of determination for EFS and OS in the chemotherapy-based studies also showed similar tendency as those in the localised osteosarcoma studies (Table 5).

**Table 4:** Correlations between surrogate endpoints and OS in patients with only localized osteosarcoma.

Surrogate endpoint	R^2^ (95% CI)	ρ (95% CI)	*P*-value for ρ	No. of studies
EFS	0.156 (0.00–0.511)	0.083 (−0.615, 0.708)	0.8312	9
1-year EFS	0.140 (0.00–0.483)	−0.067 (−0.700, 0.625)	0.8647	9
3-year EFS	0.070 (0.00–0.332)	0.367 (−0.393, 0.829)	0.3317	9
5-year EFS	0.229 (0.00–0.623)	0.377 (−0.383, 0.832)	0.3178	9
1-year OS	0.384 (0.00–0.792)	0.527 (−0.211, 0.882)	0.1447	9
3-year OS	0.453 (0.059–0.846)	0.803 (0.298, 0.957)	0.0091	9
5-year OS	0.580 (0.239–0.922)	0.767 (0.209, 0.948)	0.0159	9
RR	0.598 (0.075–1.00)	−0.800 (−0.996, 0.697)	0.2000	4

Abbreviations: CI, confidence interval; EFS, event-free survival; OS, overall survival; RR, response rate.

**Table 5:** Correlations between surrogate endpoints and OS in patients with osteosarcoma treated by only chemotherapy.

Surrogate endpoint	R^2^ (95% CI)	ρ (95% CI)	*P*-value for ρ	No. of studies
EFS	0.218 (0.00–0.625)	0.214 (−0.578, 0.798)	0.6103	8
1-year EFS	0.108 (0.00–0.436)	0.333 (−0.485, 0.841)	0.4198	8
3-year EFS	0.111 (0.00–0.441)	0.452 (−0.370, 0.877)	0.2604	8
5-year EFS	0.464 (0.057–0.871)	0.548 (−0.256, 0.904)	0.1600	8
1-year OS	0.609 (0.268–0.949)	0.826 (0.291, 0.968)	0.0114	8
3-year OS	0.371 (0.00–0.798)	0.719 (0.028, 0.945)	0.0446	8
5-year OS	0.554 (0.183–0.924)	0.762 (0.124, 0.954)	0.0280	8
RR	0.598 (0.075–1.00)	−0.800 (−0.996, 0.697)	0.2000	4

Abbreviations: CI, confidence interval; EFS, event-free survival; OS, overall survival; RR, response rate.

## Discussion

One of the advantages of using a surrogate endpoint instead of OS in clinical trials is that the required number of events can be observed more quickly; therefore, the study period can be shortened and the cost reduced. However, the concern remains whether the surrogate endpoint correctly reflects the OS, which is the true endpoint. The correlation between intermediate endpoints and OS has never been investigated in RCTs for osteosarcoma; ours is the first such investigation to date.

EFS is often selected as the primary endpoint in RCTs of localised osteosarcoma. Among the 20 RCTs analysed in our study, EFS was adopted as the primary endpoint in nine, including all of the latest four. Our results indicated that EFS might only have a moderate correlation with OS. In terms of other time-to-event endpoints, 3-year OS and 5-year OS had stronger correlations with OS than did EFS, although neither of the intermediate endpoints we investigated indicated a ‘very good’ or ‘excellent’ association with OS assessed by R^2^. The suitability of EFS and RR as intermediate endpoints for OS was not confirmed in the present study; however, if there is a need to select intermediate endpoints in RCT for osteosarcoma, 3-year OS and 5-year OS could be recommended as surrogates for OS.

Because some of the RCTs analysed in this study involved patients with metastatic disease, not all RCTs investigated localised disease only. Although the same treatment strategy is often pursued for patients with localised and metastatic osteosarcomas, their prognoses are largely different; this may result in biases when these two groups are analysed together. Therefore, a sensitivity analysis was performed using only the RCTs that investigated patients with localised osteosarcoma. This analysis revealed that the correlation between EFS and OS was inferior to that observed in the overall analysis. The same trend was observed at other time-to-event endpoints except for the 1-year EFS. The RR showed a good correlation, with an R^2^ value of 0.598; however, it was an inverse correlation (ρ = −0.800), and no surrogacy was validated. Our data showed that, even when analysing RCTs of patients with localised osteosarcoma alone, the intermediate endpoints were not found to be suitable surrogates for OS.

The fastest-obtainable surrogate endpoint is the RR. In many RCTs of patients with osteosarcoma, postoperative chemotherapy regimens were switched according to the histological responses to preoperative chemotherapy; in three of these RCTs, RR was selected as the primary endpoint^25,29,30^. Although poor responders with unfavourable RRs are known to have worse prognoses^4^, our results showed that RR was not a suitable surrogate endpoint for OS. A possible reason for this is that the outcome of osteosarcoma treatments might be influenced not only by the effect of preoperative chemotherapy but also the quality of surgery and the effect of postoperative chemotherapy. In our meta-analysis, there were six RCTs that showed significant differences in RR between treatment groups; however, none showed significant differences in OS. Even if the pathological RR is improved by increasing the intensity of chemotherapy or the number of drugs, it has not been linked to the improvement of survival^34^. This might be because the effectiveness of preoperative chemotherapy alone does not fully reflect the outcome of the overall treatment.

In the field of sarcoma, three analyses of the endpoint surrogacy were performed in patients with advanced soft tissue sarcoma (STS)^35–37^. The first found a high correlation between progression-free survival (PFS) and OS; however, only the simple correlation coefficient was calculated, whereas the coefficient of determination was not^35^. In the second study, 14 RCTs encompassing 2,846 patients with advanced STS were compiled to compare the surrogacy of intermediate endpoints for OS. Although this was the first study to collect and analyse data from individual patients with advanced STS, PFS, time-to-progression, and time-to-treatment failure were found not to be surrogate endpoints for OS in both the individual and trial levels^36^. The third study was a trial-level surrogacy validation investigation using published data; however, all RCTs used in that analysis were trials of first-line treatments for advanced STS, and the standard treatment in all RCTs was the same (doxorubicin monotherapy). The correlation between PFS and OS in that study was moderate, while the correlations of other short-term endpoints with OS were relatively poor^37^. As our analysis of RCTs of patients with localised osteosarcoma who were treated with multimodal regimens did not show strong surrogacy, and since meta-analyses of RCTs investigating patients with advanced STS treated with chemotherapy alone also failed to identify a reliable surrogate endpoint, caution should be taken when planning to use such intermediate markers as primary endpoints.

There were several limitations in our study. First of all, the analyses were based on published data and not on individual patient data; therefore, only trial-level surrogacy was analysed while individual-level surrogacy was not. Second, there were variations in the definitions of time-to-event endpoints between trials, indicating heterogeneity among included RCTs. Third, the study phases and ITT analyses were clearly described in only 6 and 7 of the 20 RCTs, respectively, while none described post-protocol treatments; this indicated that the qualities of the RCTs used in our study were not high. Finally, some trials included patients with metastatic disease (comprising 4.5% of all patients analysed); therefore, the analyses were not exclusively of patients with localised osteosarcoma.

In conclusion, our first-of-its-kind trial-level investigation of the suitability of intermediate endpoints as surrogates for OS in RCTs of osteosarcoma did not confirm their suitability, even though the rareness of the disease and the difficulty inherent in conducting large-scale RCTs were taken into account. As such, there is a need to develop novel and effective intermediate endpoints when performing RCTs for patients with osteosarcoma.

## Supplementary information

Figure S1.

Table S1.

## Data Availability

The datasets generated and/or analysed in the current study are available from the corresponding author on reasonable request.
