# Supplementary material for: Surrogate endpoints for overall survival in randomised controlled trials of localised osteosarcoma: A meta-analytic evaluation
Source: Sci Rep. 2020 May 22;10:8573. doi: 10.1038/s41598-020-65591-z (PMC7244479; doi:10.1038/s41598-020-65591-z)
Supplement: Supplementary file 1 — Figure S1. [file 41598_2020_65591_MOESM1_ESM.pdf]

Surrogate endpoints for overall survival in randomized controlled trials of localized osteosarcoma: A meta-analytic evaluation

Kazuhiro Tanaka*[1], Masanori Kawano[1], Tatsuya Iwasaki[1], Shogo Matsuda[1], Ichiro Itonaga[1], Hiroshi Tsumura[1]

[1]Department of Orthopaedic Surgery, Faculty of Medicine, Oita University, Oita, Japan

	log[HR]	SE	Weight	HR IV, Random, 95% CI
Winkler 1984	-0.2614	0.3865	1.40%	0.77 [0.36, 1.64]
Bacci 1986	0.1856	0.2752	2.50%	1.20 [0.70, 2.06]
Sweetnam 1986	-0.112	0.1658	5.70%	0.89 [0.65, 1.24]
Krailo 1987	0.131	0.1943	4.50%	1.14 [0.78, 1.67]
Burgers 1988	0.138	0.2044	4.20%	1.15 [0.77, 1.71]
Winkler 1988	-0.4308	0.2619	2.80%	0.65 [0.39, 1.09]
Bacci 1990	-0.4604	0.2547	2.90%	0.63 [0.38, 1.04]
Bramwell 1992	0.4618	0.204	4.20%	1.59 [1.06, 2.37]
Souhami 1997	0.0139	0.1378	7.30%	1.01 [0.77, 1.33]
Meyers 1998	-0.1244	0.3927	1.30%	0.88 [0.41, 1.91]
Bacci 2001	0.1398	0.2073	4.10%	1.15 [0.77, 1.73]
Goorin 2003	0.0862	0.3398	1.70%	1.09 [0.56, 2.12]
Meyers(1) 2005	0	0.1747	5.30%	1.00 [0.71, 1.41]
Meyers(2) 2005	0.2624	0.1709	5.50%	1.30 [0.93, 1.82]
Meyers(3) 2005	-0.2485	0.1839	4.90%	0.78 [0.54, 1.12]
LeDeley 2007	-0.3481	0.2076	4.10%	0.71 [0.47, 1.06]
Lewis 2007	-0.0243	0.121	8.60%	0.98 [0.77, 1.24]
Ferrari 2012	0.2577	0.2008	4.30%	1.29 [0.87, 1.92]
Bielack 2015	-0.1887	0.1559	6.20%	0.83 [0.61, 1.12]
Marina 2016	-0.0202	0.1165	8.90%	0.98 [0.78, 1.23]
Neumann 2016	0.3104	0.1846	4.90%	1.36 [0.95, 1.96]
Senerchia 2017	0.1621	0.1902	4.70%	1.18 [0.81, 1.71]
Total (95% CI)			100.00%	1.01 [0.92, 1.11]

Heterogeneity: Tau² = 0.01; Chi² = 27.64, df = 21 (P = 0.15); I² = 24%

Test for overall effect: Z = 0.28 (P = 0.78)

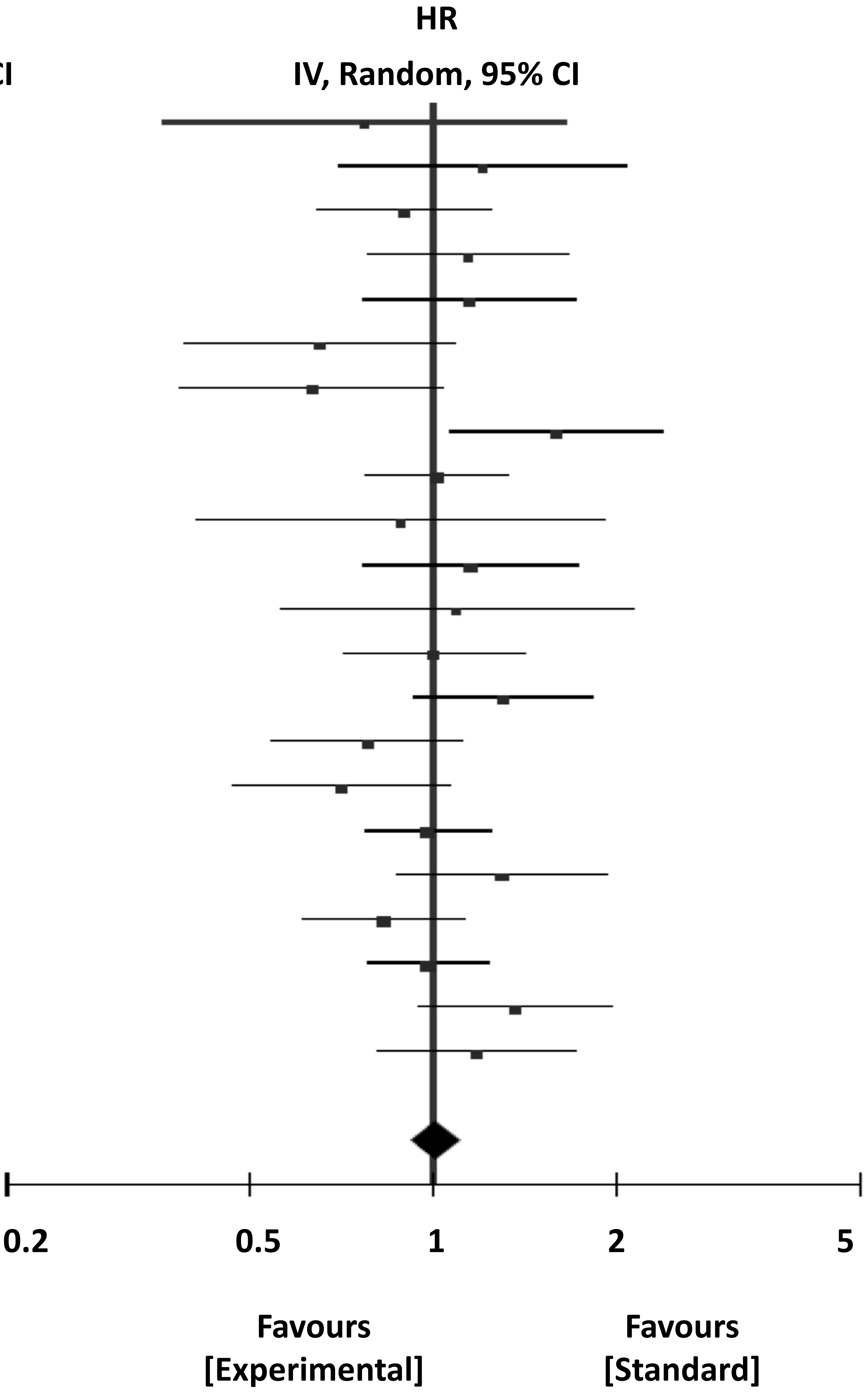

Supplementary Figure S1. Forest plot of EFS with comparison of standard vs experimental chemotherapy.

Abbreviations: CI, confidence interval; EFS event-free survival; HR, hazard ratio; IV, inverse variance; SE, standard error.
